# Comparison of whole‐head and split‐head design for the clinical evaluation of anti‐dandruff shampoo efficacy

**DOI:** 10.1111/ics.12718

**Published:** 2021-08-23

**Authors:** Yuanyuan Diao, Jane R. Matheson, Yingying Pi, Fiona L. Baines, Shuliang Zhang, Yuanpei Li

**Affiliations:** ^1^ Unilever Research and Development Centre Shanghai China; ^2^ Unilever Research and Development Port Sunlight Laboratory Bebington Merseyside UK

## Abstract

**Objective:**

Dandruff is a common scalp condition that can be improved by regular use of shampoos containing anti‐fungal actives. The efficacy of anti‐dandruff shampoos can be assessed by measuring scalp flaking, one of the important dandruff symptoms. A randomized, double‐blind trial is often used with one of two clinical designs: whole‐head parallel design and split‐head paired design. We aimed to explore the difference in product differentiation between these two designs using the same two test shampoos and the same scalp flaking assessment method (Total Weighted Head Score Adhered Flakes—TWHS AF).

**Methods:**

A clinical study was conducted with a 2‐ to 3‐week wash‐out phase and a 4‐week test phase, consisting of 2 cells: 120 subjects with whole‐head parallel design, divided into 2 subgroups (1:1) using on‐site controlled washing method (either wash their own hair at a study site, under the instruction of a study supervisor or wash their own hair at home, as per instructions, but without supervision) and 35 subjects with split‐head paired design using salon‐staff washing method. Both cells employed hair washing at frequency of three times a week and TWHS AF measurement once a week from the baseline assessment.

**Results:**

Both designs gave similar differences in TWHS AF between products: 5.6 units (95% CI: 4.1–7.0 units) in whole‐head design and 5.9 units (95% CI: 4.9–6.9 units) in split‐head design.

**Conclusion:**

Split‐head paired design shows a similar ability of detecting product difference as whole‐head parallel design, whereas it is a choice of more efficient and more cost‐effective, as only a quarter of the subjects are required to demonstrate the efficacy between anti‐dandruff shampoos.

## Graphical abstract

This study compared the efficacy of split‐head paired design and whole‐head parallel design in evaluating anti‐dandruff shampoo. As a result, split‐head paired design shows a similar ability of detecting product difference as whole‐head parallel design, whereas it is a choice of more efficient and more cost‐effective, as only a quarter of the subjects are required to demonstrate the efficacy between anti‐dandruff shampoos.

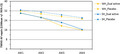

## Résumé

**Objectif:**

Les pellicules sont une affection courante du cuir chevelu qui peut être améliorée par l’utilisation régulière de shampooings contenant des principes actifs antifongiques. L’efficacité des shampooings antipelliculaires peut être évaluée en mesurant la desquamation du cuir chevelu, l’un des symptômes importants associés aux pellicules. Il est souvent fait recours à une étude randomisée et en double aveugle reposant sur l’une des deux conceptions cliniques suivantes: une conception parallèle portant sur la tête entière et une conception appariée par séparation de la surface de la tête. Nous avons cherché à étudier en quoi des produits se différenciaient entre ces deux conceptions, en utilisant les deux mêmes shampooings d’examen et la même méthode d’évaluation de la desquamation du cuir chevelu (score total pondéré des pellicules collées sur la tête [Total Weighted Head Score Adhered Flakes, TWHS AF]).

**Méthodes:**

Une étude clinique a été menée avec une fenêtre thérapeutique de deux à trois semaines et une phase d’examen de quatre semaines, composée de deux cellules: 120 sujets recrutés selon une conception parallèle portant sur la tête entière, répartis en deux sous‐groupes (1:1), avec un lavage réalisé au centre d’après une méthode contrôlée (lavage par le sujet dans l’un des centres de l’étude, réalisé sous les instructions d’un superviseur de l’étude, ou lavage par le sujet à son domicile, en suivant les instructions, mais sans surveillance) et 35 sujets recrutés selon une conception appariée par séparation de la surface de la tête, avec un lavage réalisé selon la méthode employée par le personnel des salons de coiffure. Pour les deux cellules, le lavage des cheveux avait lieu à une fréquence de trois fois par semaine et le score TWHS AF était mesuré une fois par semaine à partir de l’évaluation de référence.

**Résultats:**

Les deux conceptions ont permis d’observer des différences similaires des scores TWHS AF entre les produits: 5,6 unités (IC à 95%: 4,1 à 7,0 unités) avec la conception portant sur la tête entière et 5,9 unités (IC à 95%: 4,9 à 6,9 unités) avec la conception par séparation de la surface de la tête.

**Conclusion:**

Par comparaison avec la conception parallèle portant sur la tête entière, la conception appariée par séparation de la surface de la tête montre une capacité de détection similaire de la différence entre les produits, mais constitue un choix plus efficace et plus rentable, car elle n’exige de démontrer l’efficacité entre les shampooings antipelliculaires que chez un quart des sujets.

## INTRODUCTION

Dandruff is a common scalp condition, which is marked by scaling of the skin on the scalp. Approximately 50% of the world population will suffer from dandruff, at least sometimes in their life [1, 2]. The main symptoms vary from person to person, but they include flakes that appear on the scalp or clothing and itch or a combination of the two [3]. The aetiology of dandruff is multifactorial, but includes sebaceous lipids, *Malassezia* yeast and individual susceptibility [4]. Currently, the most common treatment for dandruff is regular use of anti‐dandruff (AD) shampoos that contain fungistatic agents, such as zinc pyrithione (ZnPT) and climbazole, or a potentiated ZnPT, which have shown significant efficacy on the resolution of dandruff [5, 6, 7, 8]. The efficacy of AD shampoos is always assessed by measuring scalp flaking, the most important dandruff symptom, within a double‐blind, randomized controlled clinical trial. Generally, there are two types of clinical study design that can be found in the literature:

- Whole‐head parallel design, with each parallel group assigned a single test shampoo and the test subjects either wash their own hair at a study site, under the instruction of a study supervisor, or wash their own hair at home, as per instructions, but without supervision [9, 10, 11, 12].
- Split‐head paired design, with just one group of subjects to compare 2 test shampoos, where the left and right sides of the head are each washed separately with a different shampoo, by trained study staff at a study site [7, 13].

It is well recognized that variation is always existing for biological endpoints, for example dandruff, which should be estimated and controlled appropriately for unbiased efficacy estimation. Generally, the random biological variation can be classified into within‐subject or intra‐individual biological variation, and the between‐subject or inter‐individual biological variation [14]. Therefore, the split‐head design, as the design of contralateral studies [15], is having each subject act as his or her own control and is able to eliminate between‐subject variation. Consequently, this may lead to a smaller sample size requirement and avoid the potential bias that caused by the confounders, which we need to control for a between‐subject design.

Theoretically, it was estimated that whole‐head design needs in approximately several times larger sample size than the split‐head design to detect an equivalent difference due to the different variation between and (or) within subjects. However, it is still unknown of the response difference in AD efficacy between the two types of design, which is more differentiable to detect products’ AD efficacy relative to controls.

Therefore, in this study, we sought to compare these two designs with the same two test shampoos and with the same scalp flaking assessment method (Total Weighted Head Score Adhered Flakes—TWHS AFs [16]), with an aim to explore the differences in product differentiation, as well to estimate the sample sizes required for the two designs of clinical AD efficacy studies in future.

## METHODS

This study was carried out in spirit of good clinical practice (GCP) guidelines and the International Conference on Harmonisation (ICH) guidelines, and in compliance with local government regulations, guidelines and standards applicable to such cosmetic clinical studies. The study was reviewed and approved by an independent ethics committee in China, and an informed consent was obtained from all study subjects. The study was registered on the ISRCTN registry with study ID ISRCTN18472520.

### Scalp flaking assessment method

Scalp flaking is assessed with the method of Total Weighted Head Score Adhered Flakes (TWHS AF) by trained expert assessors. Within this method, subjects were examined under standardized lighting conditions and successive partings were made in whole or halves of the scalp using the handle of a tail comb, depending on study design. The total area covered for each of the 6 different levels of severity (0 = none, A, B, C, D, E = most severe) is estimated using a scale of 0–10, as described in Tables 1 and 2. This could be loosely described as the percentage of area covered in 10% increments. Area of coverage estimation for each grade of severity is carried out starting from the most severe grade (E) to the completely healthy grade (0) in sequence, with all these scores adding up to 10. The estimate of the area for each grade was multiplied by the weighting score given to that severity grade condition as shown in Table 1. To calculate the TWHS AF, these scores are then added together, which gives a weighted head score. This score is then multiplied by a factor appropriate to the assessment type, to get a total weighted head score to equate to a quadrant type assessment of the whole head.

**TABLE 1:** Definition of severity grades and weighting scores for scalp adhered flaking

Severity Grade	Dandruff Description	Practical Size of Flakes	Weighting Scores
0	Perfect, healthy scalp. Uniform texture, no surface flakes	No flakes	0
A	Minimal dry powdery flakes	<0.5 mm	1
B	Small flakes at least partially adhered to the scalp. Scalp surface irregular and white	1.0 mm	2
C	Moderate flakes, loosely attached to the scalp. Scalp surface irregular and white	2.0 mm	3
D	Large pronounced flakes adhering to the scalp	2.5 mm	4
E	Very large crusting flakes. Often congealed together into yellow plates	>3.0 mm	5

**TABLE 2:** Definition of area coverage for scalp adhered flaking

Grade	Dryness/Dandruff	Particle size of flakes
0	Estimation of total area covered by each grade of severity—expressed as multiples of 1/10th units.	No loose flakes in hair with a detailed search
1–2		Minimal flakes in hair, just perceivable with a detailed search
3–4		Few loose flakes in hair, easily seen before detailed search
5–6		Moderate amounts of loose flakes in hair, obvious when looking through hair
7–8		Severe amounts of loose flakes in hair, obvious on the surface before parting hair
9–10		Excessive amounts of loose flakes in the hair, extremely obvious before parting

#### Whole‐head design

Assessment of the whole head as one single area, the calculation shown above would be done for the whole head. The weighted score is then multiplied by 4 to give a TWHS AF score on a comparable scale to the original quadrant assessment score.

#### Split‐head design

The TWHS AF scores for each side of the head need to be considered separately, and the calculation shown above would be done for each side, that is left and right. The left side would then be multiplied by 4 and the right side also by 4. This would be applicable for a subject using two different products on each half head.

The scalp visual grading of dandruff conditions by expert assessors with the TWHS method has been implemented successfully and manifested scientifically in several clinical trials published to evaluate the anti‐flaking efficacy of anti‐dandruff shampoos [7, 13, 16] and was used in the present study at baseline and weekly dandruff assessment during the four‐week treatment phase.

### Study design

This was a double‐blind (PI, subject and assessor), randomized study consisting of 2 cells: cell 1 whole‐head design and cell 2 split‐head design. Subjects were selected into cell 1 or cell 2 by randomization method and remained in the study for 6–7 weeks: a 2–3 weeks pre‐treatment wash‐out phase, followed by a 4‐week test phase.

- Cell 1 whole‐head (WH) design with on‐site controlled wash by subject: parallel design with two groups randomly assigned to one of the test shampoos
- Cell 2 split‐head (SH) design with salon‐staff wash method: paired design with the same two test products to be used at the left or right side of subject's head randomly.

**FIGURE 1:**
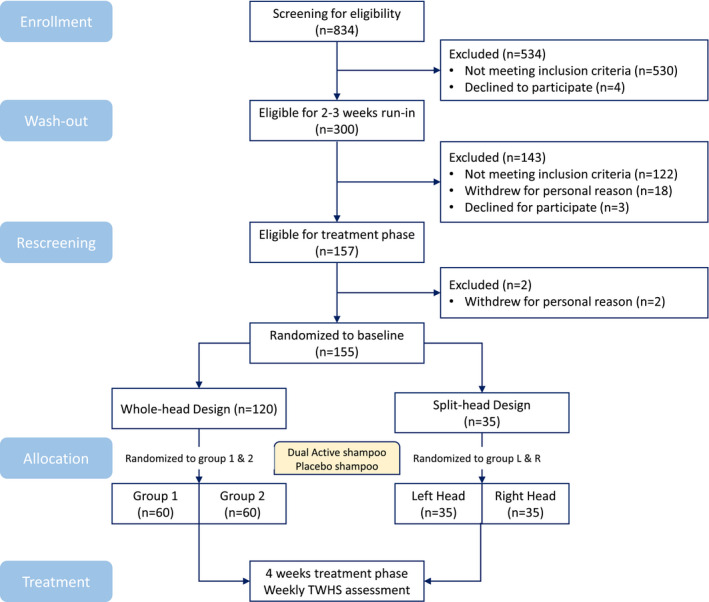
Schematic diagram of workflow

During the wash‐out period, subjects shampooed their hair three times a week at home as per normal habit using a standard marketed beauty shampoo. Those subjects who still had dandruff and met all other inclusion and exclusion criteria at the end of the wash‐out phase continued onto the subsequent test phase of the study. During the test phase, subjects in cell 1 of WH design had their hair washed three times a week at study centre by themselves with either test shampoos allocated randomly, under on‐site controlled instructions, that is follow specific instructions under salonist supervision; subjects in cell 2 of SH design had their hair washed three times a week by trained study staff at the study centre, using the randomly allocated test shampoos on each half side of head following standard half‐head hair wash procedure. The randomization allocation procedure was produced with rescreen TWHS AF and gender included as balancing factors. Dandruff was measured using the method of TWHS AF at baseline and at weekly intervals over the study. TWHS AF was assessed 48 h after the last application of shampoo. Subjects were restricted to use the shampoo they had been provided with and not to wash or wet their hair at any other time other than in the study centre for the duration of test phase. Subjects were not permitted to use any hair care products, treatments or styling products (e.g. styling gel or hairspray), neither to have their hair chemically treated (e.g. coloured, permed or chemically straightened) or hair cut at any time during the study (Figure 1).

### Study population

Healthy male and female subjects from 18 to 60 years of age who suffered from dandruff were recruited from the general population in China. Potential subjects were initially screened using a medical history questionnaire and then assessed by qualified dandruff assessors using the TWHS AF measurement to quantitate dandruff flaking severity. To be eligible to participate in the test phase of the study, subjects were required to have a TWHS AF ≥32 at the baseline visit before initiation of treatment. Subjects were excluded if they had scalp diseases; head lice/ringworm; use of anti‐inflammatory, steroids or anti‐fungal medications; history of serious illness that may require regular systemic medications; use of ketoconazole‐based shampoo within last 6 months or selenium sulphide‐based shampoo within the last 3 months or any other anti‐dandruff shampoo or hair/scalp care products within last 2 months; and any other significant medical condition.

In this study, 155 subjects were recruited into test phase and 129 subjects completed the whole study (as shown in Table 3). Totally 26 subjects were withdrawn from study, including 25 cases due to personal reasons and 1 case due to non‐compliance (hair colouring). The characteristics of the loss to follow‐up were comparable with the subjects who completed the study. There was no adverse event reported in this study.

**TABLE 3:** Study design and demographic data

Cell	Baseline age (years, M ± SD, range)	Baseline sample size	Treatment
WH Design (Group 1)	44.6 ± 11.0, 21–60	60 (28 F/32 M)	Dual‐active shampoo
WH Design (Group 2)	38.6 ± 11.3, 19–59	60 (28 F/32 M)	Negative control shampoo
SH Design	46.9 ± 10.5, 24–60	35 (15 F/20 M)	Dual‐active shampoo and negative control shampoo

### Test products

The test products used in this study were a commercially available anti‐dandruff shampoo containing dual‐active 1% zinc pyrithione (ZnPT) and 0.4% climbazole (Clear Shampoo, manufactured by Unilever), and a negative control cosmetic shampoo which is commercially available shampoo without anti‐dandruff actives (Hazeline Shampoo, manufactured by Unilever) (Table 3). As reported, clinical evaluation of the dual‐active shampoo demonstrated superior anti‐dandruff efficacy than a commercial shampoo with 1% zinc pyrithione only [7].

The test products were assigned to different groups’ subjects in cell 1 of WH design and to a different side of subjects’ head in cell 2 of SH design according to the randomization list. For the on‐site regimen, a fixed amount of test product was applied to the subject's scalp by study personnel at the clinical site three times per week on specified days of 4 weeks of test phase. After product application, each subject massaged the shampoo into their scalp and rinsed it as directed. For the salon‐staff method, a fixed amount of test shampoos was applied on each side of head and then washed by trained study staff (foaming, massaging and rinse off) at study site three times per week. Cross‐contamination was avoided by keeping the two sides parted and separating the excess water removed from the hair by a gentle blotting action with disposable paper towels.

### Statistical analysis

#### Anti‐dandruff efficacy

To compare the effects of the products over time, an analysis of covariance (ANCOVA) was conducted by an independent third‐party Statistical Services Unit (SSU), The University of Sheffield in SAS® (SAS Institute Inc., Cary, North Carolina, USA), for each of the two cells, separately. Product, time point, gender and their interactions were included as factors, along with side of head. After fitting the full model, surplus terms were removed using a backwards selection procedure (if their *p*‐value > 0.05), with the visit by‐product interaction retained to obtain estimates of interest, and baseline covariates retained in order to ensure unbiased treatment effect estimates.

#### Sample size estimation

In order to carry out prospective sample size calculations for either a WH design or a SH design, previous data from studies carried out in the same region, where dandruff was assessed using TWHS AF, were used to estimate experimental noise using the same assessors and study centre and the size of difference to detect. Separate calculations were carried out for WH and SH study designs (alpha = 0.05, power = 0.8), with separate analysis of covariance models using the individual week data as the response, baseline TWHS AF as a covariate, gender, product, visit and their interactions as fixed effects and subject ID as a random effect, to estimate the variance components and the difference between products at each week.

## RESULTS

### Anti‐dandruff efficacy (TWHS AF)

**FIGURE 2:**
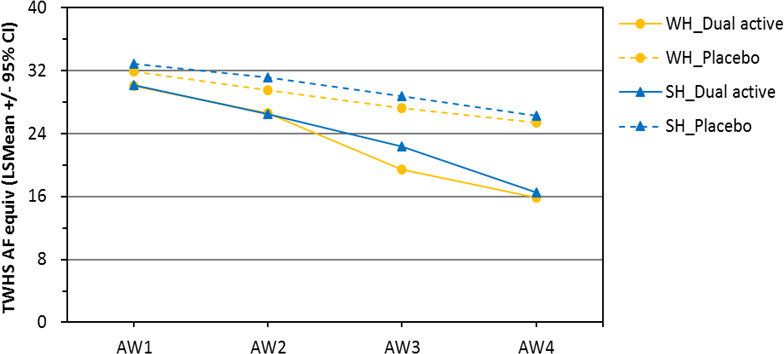
The efficiency of dual active shampoo in comparison with the control shampoo for both the whole‐head design and the split‐head design. AF, adhered flakes; SH, split‐head design; TWHS, total weighted head score; WH, whole‐head design

A significant anti‐dandruff benefit was observed for the dual‐active shampoo in comparison with the negative control shampoo (without AD actives) for both the whole‐head design (5.6 units, 95% CI: 4.1–7.0 units, *p *< 0.0001) and the split‐head design (5.9 units, 95% CI: 4.9–6.9 units, *p *< 0.0001), as shown in Figure 2.

**FIGURE 3:**
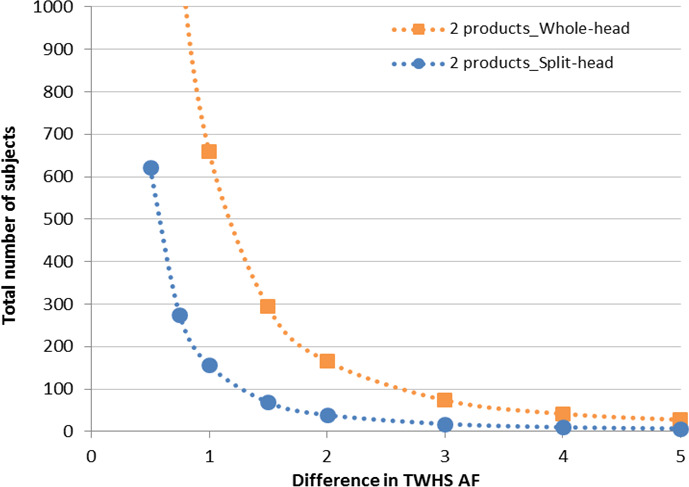
The magnitude of the difference of dual active shampoo in comparison with the control shampoo between the whole‐head design and the split‐head design. AF, adhered flakes; TWHS, total weighted head score

The estimates of product differences found in TWHS AF suggested that there was very little difference in the magnitude of the difference between products (dual‐active–negative control shampoo) between whole‐head design and split‐head design (see Figure 3). When comparing the sample size required for a new study, it seems reasonable to assume the difference to detect is the same regardless of which study design is used.

### Sample size estimation

In clinical trial, sample size estimation plays an important role for assuring validity, accuracy, reliability and integrity of the intended study. For a given study, sample size calculation is usually performed based on statistical criteria controlling type I and/or type II errors, which are conventionally defined as type I error at 5% (a = 0.05) and type II error at 10%–20% [17]. In this study, the average of variance estimated from previous whole‐head studies and split‐head studies conducted in the same region with the same population was chosen to represent the variance for whole‐head design and half‐head design, with alpha = 0.05 and desiring power = 0.8. As shown in Figure 3, the sample size was calculated for minimum base size required to compare the AD efficacy of two products corresponding to the expected TWHS AF difference for both whole‐head and split‐head designs.

**FIGURE 4:**
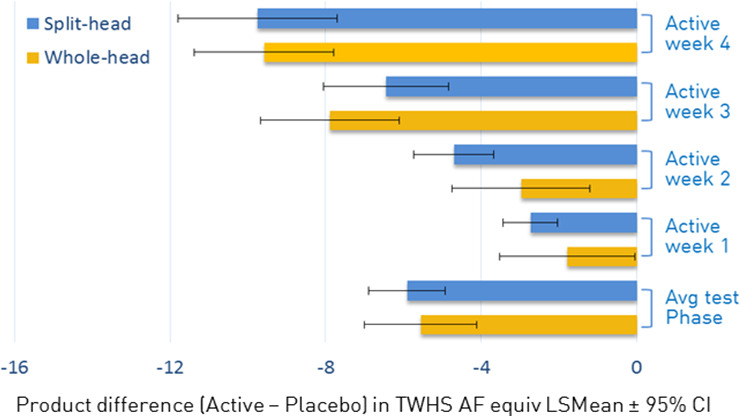
Sample size prediction of the TWHS AF and whole‐head design. AF, adhered flakes; TWHS, total weighted head score

Sample size calculations shown in Figure 4 indicate that to detect the same size of difference in TWHS AF, a whole‐head design requires more than four times as many subjects as a split‐head design with this test population. As an example, to detect a difference of 1.0 unit in TWHS AF for two products, 660 subjects are required to complete the study for a whole‐head design, whereas only 156 subjects are needed for a split‐head design.

## DISCUSSION

In this study, we have shown that both whole‐head parallel design and split‐head paired design showed a similar ability in detecting product differences in anti‐dandruff efficacy. However, it requires four times more subjects for whole‐head design than for split‐head design to detect the same size of product difference. This is consistent with the advantages of a self‐control study (or a contralateral study), as reported by Hills and Armitage [18] that if the subject can serve as his or her own control, can lead to more precise estimates for studies on the efficacy of treatments intended to reduce the frequency or severity of chronic, recurrent problems. By evaluating the same subject's dandruff condition on the left and right head with different shampoos applied on the same scalp, the variability between subjects in the frequency or severity of the problem will not hide true differences in efficacy. This can be achieved experimentally in a split‐head design also known as the half‐head study, in which one side of the scalp is treated with one shampoo and the other side with the other. This enables the comparative efficacy of the two shampoos being studied to be compared on a within‐subject basis.

Clinical study is always providing accurate and reliable assessment for the efficacy and safety of products under research. Sample size calculation plays an important role to ensure the success of studies conducted at various phases, which not only assure the study validity, but also make sure the given trials achieve a desired power for correctly detecting a pre‐specified clinically meaningful difference at a given level of significance [19]. Therefore in this paper, we estimated the sample size required for two products comparison with a whole‐head design and a split‐head design, respectively, based on the response to anti‐dandruff treatments observed from previously sufficient number of subjects using appropriate statistical methods derived under the same study design and objectives. Proper sample size can be justified based on the statistical power, clinically meaningful difference and the budget requirements.

Therefore, split‐head design shows a similar ability of detecting product difference as whole‐head design, whereas it is a choice of more efficient, more cost‐effective, as less subjects are required to demonstrate the efficacy between anti‐dandruff shampoos.
